# Differences in Ionic, Enzymatic, and Photosynthetic Features Characterize Distinct Salt Tolerance in Eucalyptus Species

**DOI:** 10.3390/plants10071401

**Published:** 2021-07-09

**Authors:** Hazar Balti, Mejda Abassi, Karl-Josef Dietz, Vijay Kumar

**Affiliations:** 1 Faculty of Sciences of Tunis, University of Tunis El Manar, Tunis 2092, Tunisia; balti_hazar@yahoo.com; 2 Department of Biochemistry and Physiology of Plants, Faculty of Biology, University of Bielefeld, 33615 Bielefeld, Germany; vijay.kumar@uni-bielefeld.de; 3 Laboratory of Forest Ecology, National Research Institute of Rural Engineering, Water and Forests, Street Hedi Elkarray, Elmenzah IV, BP 10, Ariana 2080, Tunisia; mej_abassi@yahoo.fr

**Keywords:** Eucalyptus, salinity, photosynthesis, photosystem II, photosystem I, plastocyanin, ferredoxin, redox, osmotic balance, hydrolases

## Abstract

In the face of rising salinity along coastal regions and in irrigated areas, molecular breeding of tolerant crops and reforestation of exposed areas using tolerant woody species is a two-way strategy. Thus, identification of tolerant plants and of existing tolerance mechanisms are of immense value. In the present study, three Eucalyptus ecotypes with potentially differential salt sensitivity were compared. Soil-grown Eucalyptus plants were exposed to 80 and 170 mM NaCl for 30 days. Besides analysing salt effects on ionic/osmotic balance, and hydrolytic enzymes, plants were compared for dynamics of light-induced redox changes in photosynthetic electron transport chain (pETC) components, namely plastocyanin (PC), photosystem I (PSI) and ferredoxin (Fd), parallel to traditional chlorophyll a fluorescence-based PSII-related parameters. Deconvoluted signals for PC and Fd from PSI allowed identification of PC and PSI as the prime salinity-sensitive components of pETC in tested Eucalyptus species. *Eucalyptus loxophleba* portrayed efficient K^+^-Na^+^ balance (60–90% increased K^+^) along with a more dynamic range of redox changes for pETC components in old leaves. Young leaves in *Eucalyptus loxophleba* showed robust endomembrane homeostasis, as underlined by an increased response of hydrolytic enzymes at lower salt concentration (~1.7–2.6-fold increase). Findings are discussed in context of salinity dose dependence among different Eucalyptus species.

## 1. Introduction

Soil salinity describes the accumulation of soluble inorganic cations such as Na^+^, Mg^2+^ and Ca^2+^, together with anions in the form of chloride, carbonate, or sulfate [1]. In fact, excessive use of ion-rich irrigation water causes soil salinization, and this effect is particularly severe in arid and semi-arid regions. Salinity is a major form of abiotic stress that affects plant growth, development, and agriculture productivity worldwide [2,3]. Current estimates suggests that >6% of the world’s land and >30% of all irrigated areas are already suffering from exposure to salinity [4]. In addition to crop yield losses, salinity-dependent vegetation loss has other severe negative impacts on the local ecosystem, such as the removal of natural CO_2_ sinks with adverse consequences on climate change, soil erosion, poor water retention, etc. These impacts further accentuate drought and salinity effects [5,6,7,8]. Reforestation has been a strategy used to counter such self-reinforcing processes. Fast-growing and salinity-tolerant tree species have shown potential to provide soil stabilization with wood production and could be a long-term solution to restore vegetation in marginal areas and sites affected by salinity [9,10,11,12]. For example, Eucalyptus, a multipurpose woody species that develops an extended deep root system, is suitable for many landscape applications including reclamation of dry arid saline lands. In fact, the area under Eucalyptus cultivation has increased 30 times in roughly 60 years, especially in tropical and subtropical countries [13]. Many Eucalyptus species like *E. sargentii*, *E. camaldulensis*, *E. loxophleba*, *E. spathulate*, *E. agrophloia* and *E. neglecta* show capacity to tolerate salinity [14,15,16] and have been used widely to explore salinity tolerance mechanisms in plants.

Salinization leads to reduced soil fertility and decreased water availability [3]. Therefore, along with the accumulation of cytotoxic ions, nutritional imbalance and osmotic and oxidative stresses are major challenges for plants facing salinity [3,17]. Plants use many complementary strategies to counter the negative effects of salt stress [18]. Accumulation of Na^+^ and Cl^−^ ions in the cytosol, stroma, and matrix, the so-called “plasmatic compartments” of the cell, has a chaotropic effect on structure and function of cell membranes and proteins [17,19]. Therefore, ion compartmentation by deposition in the vacuole, often of certain cell types like trichomes, epidermis and bladder cells, allow for the lowering of ion concentrations in the plasmatic compartments [17,20,21]. Inadequate compartmentation causes ion accumulation in plasmatic compartments and consequently the loss of membrane integrity, inhibition of activities of different metabolic enzymes and altered cell signaling.

The exclusion of detrimental ions from the cytoplasm is realized by strict regulation of transport processes and synthesis of compatible solutes [20,22,23]. Overall, ion homeostasis is a delicate interaction between inter-organ, organ, inter-cellular and subcellular transport processes [24]. For vacuolar transport and subsequent osmotic homeostasis, ion/H^+^-antiporter, vacuolar Cl- channels and tonoplast intrinsic proteins have been implicated [17,24,25]. Despite widely emphasized involvement of vacuoles in ion homeostasis under salinity, the regulation and effects of the accumulated ions on the crucial proteolytic hydrolases, phosphatases or phosphoesterases have not been addressed [17,21]. It is important to note that the regulation of ion accumulation in different compartments depends on factors like total external ion concentrations, effective osmotic homeostasis mechanisms, prevalence of counter-anions like K^+^, or Ca^2+^ transport etc. [17,24]. For example, K^+^ concentrations need to be kept high for proper enzyme function and translation, while sodium inhibits these processes [26,27]. For this reason, maintenance of a high potassium-to-sodium ratio under salinity in plasmatic compartments of plant tissues, despite being energy intensive, is crucial for proper activities of several enzymes and membrane functions [17,22,28].

Salinity effects on plant growth can be partially linked to inhibited photosynthesis. Effects of salt exposure on photosynthesis are realized in two stages, where the early osmotic shock and later ion accumulation are responsible for widely observed negative interferences with yield [20]. Perturbed water uptake due to reduced soil water potential leads to osmotic stress, which in turn affects transpiration, stomatal function, and gaseous exchange, thus reducing internal CO_2_ levels [20]. These changes influence regulation of RuBisCO activity resulting in inhibited carbon assimilation [29,30]. Ionic imbalance, on the other hand, majorly affects optimal protein function in the photosynthetic electron transport chain (pETC), the light dependent photosystem II (PSII; P_680_) damage-repair cycle, and the efficient generation of redox metabolites and excessive generation of reactive oxygen species (ROS); hence, the result is oxidative stress [20,29,31]. Several studies have reported, e.g., inhibition of effective photochemical yield (ɸPSII), PSII activity, electron transport rate (ETR), photochemical quenching (F_v_/F_m_, qP), and CO_2_ assimilation upon exposure to salinity [2,20,32,33]. In successful acclimation to salinity stress, the optimal control of activity of ROS-generating enzymes like plasma membrane-situated NADPH oxidases (RBOH), mitochondrial alternate oxidase (AOX), and plastid terminal oxidase (PTOX) along with ROS scavenging enzymes like superoxide dismutase, catalase, or several types of peroxidases have been considered essential [33,34].

PSII efficiency has been long correlated to carbon assimilation under abiotic stresses in general, or specifically under salinity. However, the interrelationship between regulation of stress acclimation and the effects of stresses on the efficiency of other components in pETC remain largely unexplored, which is focus of this study. As described above, studied salinity effects on photosynthesis have been mostly observed on parameters related to activity, the damage repair cycle of PSII, and, to some extent, on net carbon assimilation rates [21]. On the other hand, redox changes, activity, reaction efficiency and changes in relative pool size of other pETC components on the acceptor and donor sides of PSI (P_700_) like plastocyanin (PC) and ferredoxin (Fd) have not been analysed before. One significant reason for this was unavailability of specific, non-invasive analytical methods that could distinguish light absorption/fluorescence characteristics of components like PC, PSI and Fd [35,36]. The recently developed kinetic LED array spectrophotometer (DUAL-KLAS-NIR, Walz, Germany) allows such deconvolution to quantify light-induced redox changes in PC, PSI, and Fd in intact leaves [35,36,37]. Besides being rapid and highly accurate, these non-invasive procedures facilitate comparisons of stress effects among plants with different leaf morphology, as the quantification is not impaired due to, e.g., different chlorophyll contents or stomatal density. Another major advantage here is that a parallel measurement for chlorophyll a fluorescence is also possible, which allows comparison of stress effects among different pETC components (PSII and PSI) as has been already illustrated in several recent studies for diverse abiotic stresses [38,39,40,41,42].

Thus, in the current study three Eucalyptus species were selected to analyze their salt stress response. The specific objective was to identify overlapping and adaptive changes based on traditional features such as growth, osmotic relations, ion accumulation and oxidative stress. These data were complemented by non-invasive and parallel measurements of efficiency and redox shifts for the pETC components PSII, PC, PSI and Fd. The results underline the inter-relationship between the measured parameters for salt stress acclimation through differential effects among salt-tolerant and -sensitive species and suggests utility and applicability of these non-invasive measurements in salt tolerance assessment.

## 2. Results

### 2.1. Plant Morphology and Biomass Production

The selection of plants to be analysed in the current study was based on the known salt tolerance versus sensitivity of Eucalyptus species. However, to account for differences in salt tolerance that could exist for ecotypes, we also considered the outcome of a preliminary screen of germination and seedling growth involving six Eucalyptus species growing in forests in Tunisia.

This screening assessed the responses of *E. gomphocephala* A. Cunn. ex DC., *E. sargentii* Maiden, *E. loxophleba* Benth., *E. gillii* Maiden, *E. torquata* Luehm., and *E. gracilis* F. Muell. (Supplementary Materials Tables S1 and S2). Thus, during early seedling stages selected species *E. loxophleba* and *E. gomphocephala* showed similar tolerance to NaCl (0–210 mM NaCl), while *E. torquata* appeared salt-sensitive even at lower salt concentrations (Supplementary Materials Tables S1 and S2). The results of the germination study prompted us to select a moderate (80 mM) and high (170 mM) salt exposure in adult plants for the main experiment.

**Figure 1:**
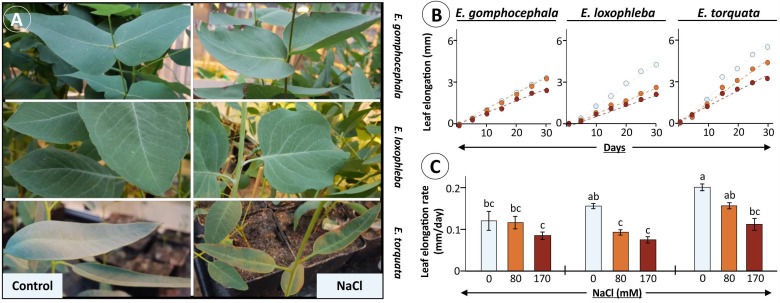
Leaf growth phenotype for Eucalyptus species under salt stress. Selected Eucalyptus species were exposed to increasing amounts of NaCl (0, 80 and 170 mM) and observed for leaf growth rates during 30 d of treatment. (**A**) Leaf phenotype as photographed after 30 d of maximum salt exposure to 170 mM. Measured leaf elongation presented as a growth-time curve (**B**) during treatment period along with calculated leaf elongation rate per day (**C**) for each species. Data for elongation rate per day (**C**) are means ± SE (*n* = 8, *p* < 0.05; ANOVA and Tukey’s post-hoc test).

In general, growth comparison for these three Eucalyptus species in terms of total biomass accumulation under optimal growth conditions for a year revealed highest growth rates for *E. torquata* (Et) (Supplementary Materials Figure S1). In 6-month-old and subsequently 30 d salt (170 mM)-exposed plants, leaves of *E. torquata* developed necrotic spots that were not so prominent in the other two species. Further, *E. gomphocephala* (Eg) leaves also showed substantial leaf curling, which was much less in *E. loxophleba* (El) (Figure 1A). No visible differences were observed in leaf phonotype for all three species at the lower salt concentration of 80 mM. Chlorophyll and carotenoids contents showed a similar pattern with a significant decline for both in *E. torquata* leaves after prolonged salt exposure (Supplementary Materials Figure S2A–D).

**Figure 2:**
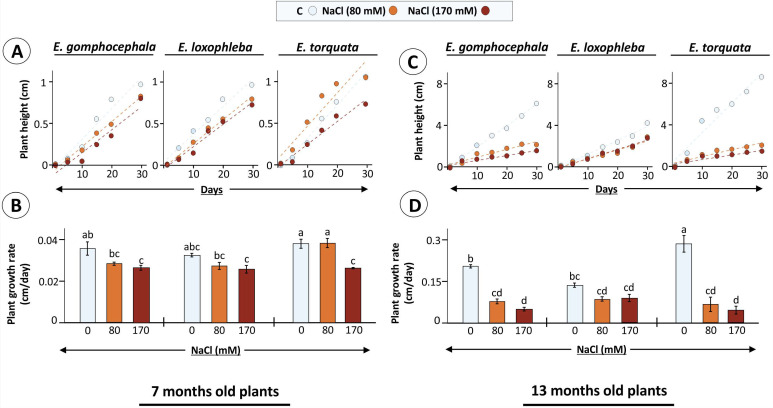
Eucalyptus plant growth as influenced by salt treatment. Eucalyptus species were exposed to NaCl (80 and 170 mM) added to the growth substrate after 6 (**A**,**B**) or 12 (**B**,**C**) months after germination and their growth was recorded during next 30 d. In (**B**,**D**), data are means of plant growth rate ± SE (*n* = 3–4, *p* < 0.05; ANOVA and Tukey’s post-hoc test; bar diagrams).

Further analysis revealed differences in salt stress effects also on leaf elongation in tested Eucalyptus species (Figure 1B,C). Significant reduction in leaf elongation rate was observed for *E. loxophleba* (41 and 53% at 80 and 170 mM NaCl, respectively), and *E. torquata* (45% at 170 mM), while the decrease was minimal in *E. gomphocephala* (Figure 1C). Notably, the total rate of leaf elongation was higher for *E. torquata*, like the biomass accumulation, even after 30 d salt exposure. This was also the case for plant growth rates for both 7-month and 13-month-old plants (Figure 2A–D).

Data suggests that salt stress effects were plant age-dependent, where 7-month-old plants showed a 0–31% decrease in plant growth rate after 30 d salt exposure. Incidentally, the minimum (no change; 80 mM NaCl) and maximum decrease (31%; 170 mM NaCl) was observed for E. torquata (Figure 2B). The other two Eucalyptus species showed a steady decrease (~20%) already at 80 mM treatment. Similarly, salt exposure for 30 d had more pronounced effects on 13-months-old plants, where treatment-specific decreases in plant growth rate were 62 and 76 (Eg), 36 and 34 (El), and 76 and 84% (Et) for 80 and 170 mM NaCl, respectively (Figure 2D). These observations point to growth responses as indicators of plant-specific stress tolerance mechanisms.

### 2.2. Osmotic Balance and Ion Homeostasis in Plants under Salinity Stress

**Figure 3:**
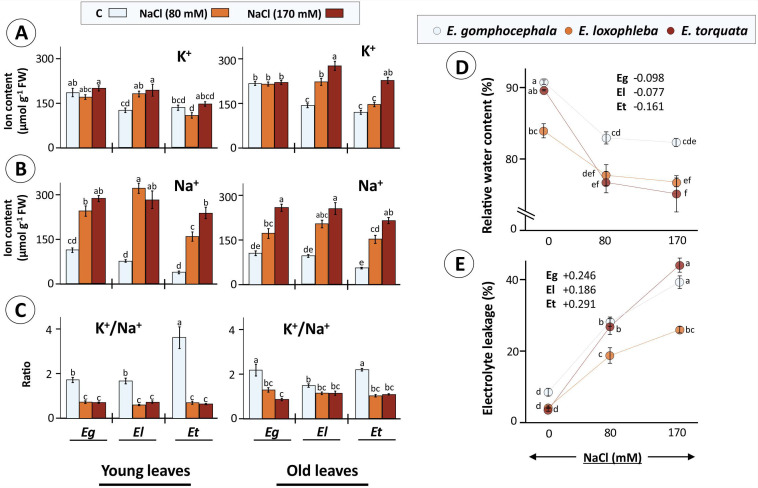
Osmotic and ionic burden as faced by Eucalyptus grown in saline conditions. Eucalyptus spp exposed to salinity (80 and 170 mM NaCl) experienced both osmotic stress as well as ionic imbalance which was measured as tissue K^+^ (**A**) and Na^+^ (**B**) contents in young and old Eucalyptus leaves and their ratios (**C**) as well as relative water content (RWC) (**D**) and membrane integrity (electrolyte leakage; EL) (**E**). RWC and EL scatter plots also provide values for slopes for each species indicating NaCl effects (only 80 mM as effect saturates for 170 mM). Data are mean ± SE, *n* = 4 (RWC, EL) and 5 (ion contents). Ca^2+^ contents are given in Supplementary Materials Figure S3A,B. Significant differences are based on ANOVA with Tukey’s post-hoc test (*p* < 0.05).

In the present setup, ion accumulation (K^+^, Na^+^, and Ca^2+^) was measured in young and old leaves of each Eucalyptus species and showed leaf age-dependent variations (Figure 3A–C; Supplementary Materials Figure S3). Salinity treatment led to increased K^+^ tissue contents in *E. loxophleba* young leaves (49 and 59%) and old leaves (60 and 97%) under 80 and 170 mM NaCl, respectively. In comparison, no significant change was observed for K^+^ ions in *E. gomphocephala* and *E. torquata*, except for a 93% rise for old Et leaves at 170 mM (Figure 3A). Increased tissue K^+^ serves to counterbalance accumulated Na^+^ and maintain a functional K^+^/Na^+^ ratio. Measured Na^+^ amounts revealed a prominent treatment-dependent accumulation in old leaves of all three Eucalyptus species, i.e., 1.64-, 2.47-fold (Eg), 2.12-, 2.64-fold (El), and 2.75-, 3.93-fold (Et) under 80 and 170 mM NaCl, respectively (Figure 3B). In contrast, in young leaves in both El and Eg, both treatments led to a similar increase of 2- and 4-fold, respectively, while the increase in Na^+^ accumulation for Et was significantly different (4- and 6-fold) for increased NaCl-treatments (Figure 3A). Due to changes in K^+^ and Na^+^ accumulation with salinity, the K^+^/Na^+^ ratio declined in both young (60% for Eg, El and 80% for Et) and old leaves (40–60% in Eg, 50% in Et but only 25% in El) (Figure 3C).

The relative water content (RWC) is an indicator of the water deficit encountered by the plants under salinity. Leaf RWC ranged between 90–70%, where plants subjected to salt stress (80 and 170 mM NaCl) showed significantly reduced RWC (Figure 3D). The degree of RWC decrease was higher for *E. torquata* as was also evident from a maximum 16% drop in RWC for Et as compared to 9% in both El and Eg, respectively, for 170 mM NaCl treatment (Figure 3D). EL, on the other hand, is a marker of plant plasma membrane integrity which is perturbed by ionic accumulation in plasmatic compartments and subsequent ROS accumulation. *E. gomphocephala* and *E. loxophleba* plants showed a 3- to 6-fold higher EL for the salt-treated leaves, but in comparison the leaves of *E. torquata* had 7- and 11-fold higher EL under 80 and 170 mM NaCl, respectively (Figure 3E).

### 2.3. Photosynthesis and pETC: Working of PSII and PSI under Salinity Stress

**Figure 4:**
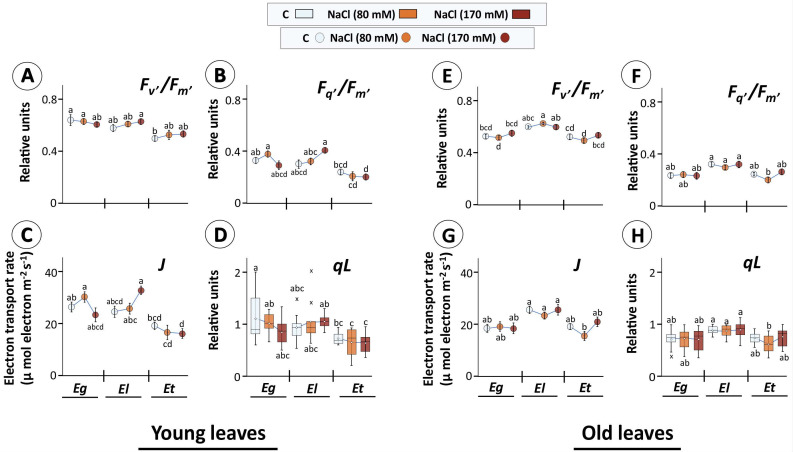
Efficiency of photosystem II (PSII) in selected Eucalyptus species exposed to salinity. Dark-adapted, detached young (**A**–**D**) and old leaves (**E**–**H**) of 30 d salt treated Eucalyptus species were exposed to actinic light (160 µmol m^−2^ s^−1^) for 17s and chlorophyll a fluorescence recorded using the LED array spectrophotometer (DUAL-KLAS-NIR, Walz, Germany). Different parameters like maximum PSII efficiency in light (*F_v’_/F_m’_*) (**A**,**E**), operating PSII efficiency in light (*F_q’_/F_m’_*) (**B**,**F**), electron transport rate (ETR; *J*) (**C**,**G**), and estimated fraction of open PSII reaction centers (*qL*; data given in form of box plots; (**D**,**H**)) were calculated from chlorophyll a fluorescence data (means ± SE, *n* = 9–12, *p* < 0.05; ANOVA and Tukey’s post-hoc test). (In box plots: hollow circle = mean, the horizontal line in the box = median, box limits = 25th, 75th percentiles, whiskers extend 1.5 times the interquartile range from the 25th and 75th percentiles, not connected data points; cross = outliers).

As noted above, salinity exposure in the present setup reduced chlorophyll as well as carotenoid contents with maximum change observed for *E. torquata* under both treatments. A similar pattern was observed for chlorophyll fluorescence-based parameters, especially in young leaves (Figure 4A–D). Overall, all parameters calculated showed the lowest values for *E. torquata*, where the operating PSII efficiency in light (Fq’/Fm’) (Figure 4B) and ETR (Figure 4C) decreased further with salt treatments, as was also the case for qL (Figure 4D); however, this showed larger variability. The Fq’/Fm’ and ETR values showed similar patterns, as ETR is calculated based on Fq’/Fm’. In contrast to Et, Fq’/Fm’, Fq’/Fm’ and ETR all had an increasing trend with salt treatment for *E. loxophleba* (Figure 4A–D).

In old leaves, however, the values for all parameters were highest for El, without any apparent effect of salt treatment in El and Eg (Figure 4E–H). In Et old leaves, 80 mM NaCl reduced the values for all parameters consistently, however they increased again for 170 mM treatment. It needs emphasis that old leaves were those that started to grow before start of the treatment period, while young leaves originated by growth during one month of salt exposure. Further, one reason for observed uniformly low values for operating PSII efficiency in light might be the 90 min dark adaptation in between the light period (Figure 4).

**Figure 5:**
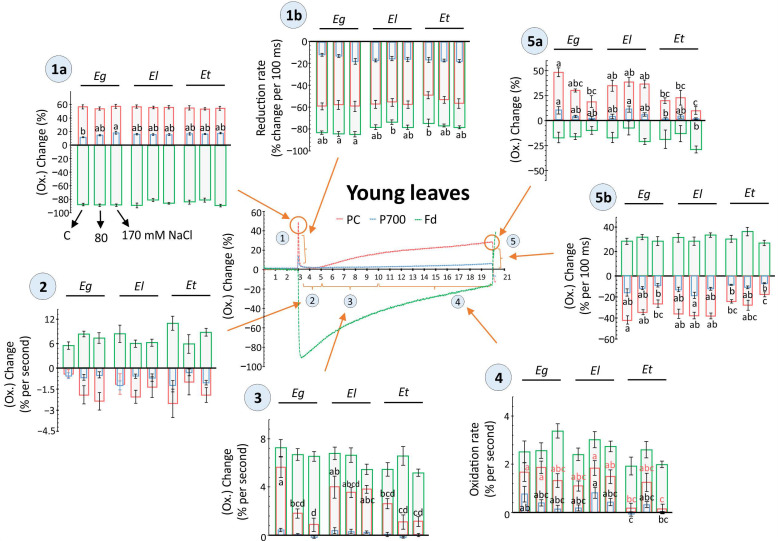
Light-driven redox changes for PC, P700 and Fd as recorded in young leaves of Eucalyptus spp under salt stress. Dark-adapted, detached young leaves of 30 d salt treated Eucalyptus spp were exposed to 160 µmol m^−2^ s^−1^ for 17 s and redox states of plastocyanins (PC), photosystem I (PSI) and ferredoxin (Fd) were recorded along with chlorophyll a fluorescence (Figure 4) using the DUAL-KLAS-NIR (Walz, Germany). The scatter plots depict representative kinetics of light-driven redox change at five different phases, as indicated i.e., 1(a, b), 2, 3, 4, 5 (a, b), based on light-dependent temporal shifts in the redox state of PC, PSI, and Fd (Phase 1 = Dark to light shift, Phase 2, 3 = dynamics of reoxidation, Phase 4 = apparent steady state, Phase 5 = end of light phase and light to dark shift. In phase 1 and 5, ‘a’ = max. value, ‘b’ = rate of redox shift). For individual phases, the redox changes were compared among plant species and different salt treatments and shown as histograms in form of the maximal changes or rates of redox transition (means ± SE, *n* = 9–12, *p* < 0.05; ANOVA and Tukey’s post-hoc test).

**Figure 6:**
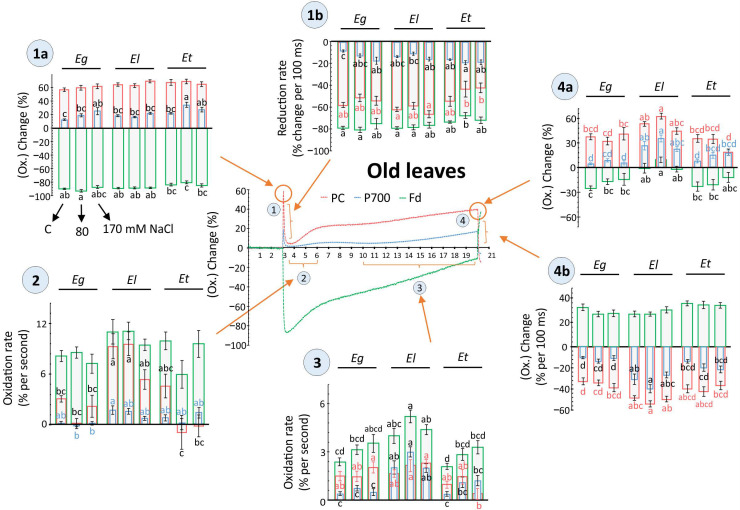
Light-driven redox changes for PC, P700 (PSI) and Fd as recorded in old leaves of Eucalyptus spp under salt stress. Dark-adapted, detached old leaves of 30 d salt treated Eucalyptus spp were exposed to 160 µmol m^−2^ s^−1^ for 17 s and redox states of plastocyanins (PC), photosystem I (PSI) and ferredoxin (Fd) were recorded along with chlorophyll a fluorescence (Figure 4) using the DUAL-KLAS-NIR (Walz, Germany). The scatter plot showing representative kinetics of light-driven redox change was divided into four different phases, as indicated i.e., 1(a, b), 2, 3, and 4(a, b), based on light-dependent temporal shifts in the redox state of PC, PSI, and Fd (Phase 1 = Dark to light shift, Phase 2, 3 = dynamics of reoxidation and apparent steady state, Phase 4 = end of light phase and light to dark shift. In phase 1 and 4, ‘a’ = max. value, ‘b’ = rate of redox shift). For individual phases, the redox changes were compared among plant species and different salt treatments and are shown as histograms in form of maximal change or rate of redox transition (means ± SE, *n* = 9–12, *p* < 0.05; ANOVA and Tukey’s post-hoc test). The data between 6–10 s were excluded due to observed high variance.

The efficiency of PSI and hence downstream carbon assimilation steps are not only determined by the activity or state of PSII, but by the balance between the pool sizes and the rate of reduction/oxidation of the acceptor (PC) and donor side (Fd) components for the P700 reaction center. In young Eucalyptus leaves, no major change in the maximum redox shift and pool size for PC, PSI and Fd was observed, except for the 80 mM NaCl treatment where the Fd response in Et was decreased compared to its control as well as that observed for El under 170 mM salt (Supplementary Materials Table S3). In old leaves, maximum redox changes for PC and its pool size ratios relative to PSI were strongly lowered in *E. torquata*, especially under 170 mM salt treatment (Supplementary Materials Table S3). Under the same treatment, the maximal redox change for PSI was highest for *E. torquata*. Further, light-dependent multiphasic redox changes recorded for PC, PSI and Fd for both young and old leaves are given in Figure 5 and Figure 6. The evaluation considered multiple phases based on light-dependent electron flow upon dark-to-light transition, the rapidity of activation of reactions downstream to Fd, the redox state in the steady state of light-driven reactions as well as the rapidity of redox change for the final light-dark.

Major differences in redox state or respective rate of change were observed for old leaves, where more than one analyzed component, i.e., PC, PSI or Fd, showed a salt- or species-dependent variation. Also, among all compared phases of the redox kinetics in both young and old leaves, dark-to-light transitions or vice versa and the apparent steady state phase showed major differences (Figure 5 and Figure 6). Further, both in young and old leaves, the acceptor side of PSI revealed more pronounced differences than the donor side (Figure 5 and Figure 6). For example, in young leaves, the rate of PC reoxidation after 2 s of light exposure was strongly impeded by both salt treatments in Eg and Et, but not in El (Figure 5, phase 3). Similarly, rapid PC reduction upon darkening was lowered by salt exposure in Eg and Et, but especially in Et by 170 mM NaCl (Figure 5, phase 5b). PC and PSI reoxidation rate and maximal values after reaching apparent steady state at 17 s of light exposure were also decreased in Et, however effects were not salt concentration-dependent (Figure 5, phase 4 and 5a).

In old leaves, at the onset of light, maximum Fd reduction was decreased, while PSI oxidation was increased in Et plants treated with 80 mM salt compared to others (Figure 6, phase 1a). Following the initial oxidation peak, PC reduction was strongly impeded by both salt treatments (Figure 6, phase 1b). Again, in the next reoxidation step, the rate for PC was lowered in Et, compared to El, especially at 80 mM NaCl (Figure 6, phase 2). In the next slower reoxidation phase, the rate for PC was also lower under maximum salt treatment in Et (Figure 6, part 3). In the same phase, Fd oxidation rates were higher for El, compared to control values in Eg and Et, while in all Eucalyptus spp salt had stimulating effects on Fd oxidation (Figure 6, phase 3). Maximum values at the apparent end of light phase were higher for all the components (PC, PSI, Fd) for El compared to Eg and Et, while 170 mM NaCl decreased the maximal values for PC and Fd in Et (Figure 6, phase 4a). Similarly, higher rates of reduction were observed for El upon darkening, with no major effects of salt treatment (Figure 6, phase 4b).

### 2.4. Activities of Hydrolases

**Figure 7:**
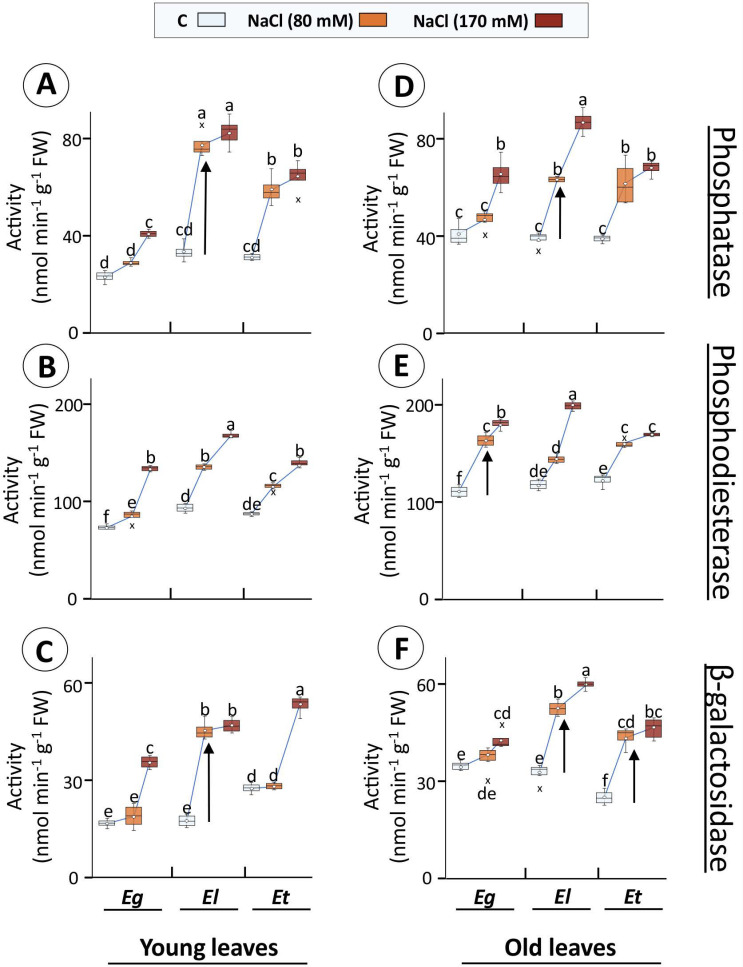
Activities of hydrolases in Eucalyptus leaf extracts in dependence on salt exposure. Activities of phosphatase (**A**,**D**), phosphodiesterase (**B**,**E**), and β-galactosidase (**C**,**F**) were measured in young and old leaves of 30 d salt (80 or 170 mM NaCl)-treated Eucalyptus spp. Different letters indicate significance of difference after statistical analysis of the means (*n* = 4, *p* < 0.05; ANOVA and Tukey’s post-hoc test). (In box plots: hollow circle = mean, the horizontal line in the box = median, box limits = 25th, 75th percentiles, whiskers extend 1.5 times the interquartile range from the 25th and 75th percentiles, not connected data points; cross = outliers).

The phosphatase activity in young leaves was significantly higher for Eg (76%) only at 170 mM treatment, while it was 2.33, 2.47-fold and 1.89, 2.07-fold for El and Et, respectively, with increasing salt treatment (Figure 7A). In the old leaves, however, all three species showed a different behaviour, where for Eg the increase was significant only for 170 mM (61%), for Et the change was similarly higher for both treatments (~1.65-fold), and old El leaves displayed a treatment specificity with 1.64- and 2.24-fold increase at 80 and 170 mM NaCl, respectively (Figure 7D). In the case of phosphodiesterase, in young leaves of all species, the increase in activity was treatment-dependent. Thus, a 17, 82% (Eg), 45, 81% (El) and 33, 62% (Et) increase was observed at 80 and 170 mM, respectively (Figure 7B). Conversely, the pattern of increase differed among the Eucalyptus species in old leaves (Figure 7E). Here, Eg showed treatment specificity with an increase of 48 and 64%, while for El the increase was only significant at 170 mM (70%). In *E. torquata*, the increase was similar for both treatments (~34%) (Figure 7E). β-galactosidase activity also increased differently among Eucalyptus species and leaf age types. For example, in young leaves of Eg (2.14-fold) and Et (1.93-fold) the only increase was for 170 mM NaCl treatment, while in the same case for El, both treatments led to a similar increase (~2.65-fold) (Figure 7C). On the other hand, in old leaves, the increase was 25% (Eg; 170 mM), ~80% (Et; both treatments) and a treatment-specific 61 and 84% (El; 80 and 170 mM, respectively) (Figure 7D).

In addition, notably higher constitutive catalase activity was observed in El and Eg compared to Et both in young and old leaves (Supplementary Materials Figure S4A,B). Catalase activity was significantly reduced in young (by 81, 86% for 80 and 170 mM NaCl, respectively) and old leaves (by 71, 79% for 80 and 170 mM NaCl, respectively) of *E. loxophleba*, whereas the only significant decrease (75%) for *E. gomphocephala* occurred in old leaves at 170 mM NaCl (Supplementary Materials Figure S4A,B). However, no significant change for catalase activity was observed for *E. torquata* on salt exposure.

## 3. Discussion

In plants, efficient salinity tolerance strategies involve complementary mechanisms at organ, cellular and subcellular levels. Examples for such crucial mechanisms are maintenance of osmotic balance through accumulation of osmolytes, compatible ion accumulation, protection of enzyme and membrane function in compartments, adjustment of redox balance, and the protection of photosynthetic machinery against oxidative damage as well as regulation of growth rates [2,17]. In the present setup, three Eucalyptus species which apparently possess differential salinity tolerance were compared. Eucalyptus plants are native to coastal regions and hence many species possess a natural ability to tolerate salinity [14]. The salt tolerance and sensitivity for *E. loxophleba* and *E. torquata* growing in different regions, respectively, are well documented features [15,43], while *E. gomphocephala* is a known coastal species and possesses drought tolerance traits [44].

Salt exposure studies in Eucalyptus have shown significant modulation of plant height and biomass accumulation in *E. camaldulensis*, *E. sargentii*, and *E. loxophleba* among other woody species [15,45]. Recorded phenotype, like appearance of necrotic spots and leaf browning and curling in *E. gomphocephala* and *E. torquata* indicated severe salt-induced damage at cellular level, especially in the latter (Figure 1A). Interestingly, continued high growth rates during 30 d salt treatment for 6-months old *E. torquata* plants could hint at inability to adjust balance between energy expenditure for growth and defence, which was efficiently portrayed by *E. loxophleba* plants (Figure 1B and Figure 2A). This hypothesis is supported by the constant 20–30% decrease in growth for 7- or 13-month-old El plants under salinity, while the same was either 30 or >80% for Et, respectively (Figure 2). Successful salt tolerance includes three mechanisms: (1) prevention of damage, (2) establishment of homeostasis and (3) adjustment of sustainable growth [46]. Such phenotypic plasticity is provided by various hormonal signaling pathways, along with the salt overly sensitive (SOS) signaling pathway [47,48,49]. This could indicate a regulated allocation of resources in El for management of the ionic/osmotic challenge, maintaining photosynthetic efficiency and prolonged survival under prevailing saline conditions for salt-tolerant plants as discussed below.

### 3.1. The Osmotic and Ionic Challenge

Salinity inhibits plant growth via ionic imbalance and subsequent disruption of cellular metabolism [50]. The SOS signaling pathway has an established role in ion-homeostasis and regulates cellular Na^+^ contents through extrusion and compartmentalization [49]. Recently, the sucrose non-fermentation-related protein kinases (SnRKs) of subgroup SnRK3, which participate in SOS signaling pathways, were characterized also in Eucalyptus species (*E. grandis*) [51]. Presented data from Tunisian Eucalyptus ecotype of *E. torquata* clearly indicated lack of regulation in Na^+^ uptake or extrusion from roots as cellular Na^+^ load increased in young and old leaves alike with increasing salt exposure (Figure 3A–C). Similar findings were reported for *E. globulus*, a species with greater susceptibility to salinity, as plants were unable to exclude Na^+^ [14]. In contrast, Na^+^ amounts in young leaves of *E. loxophleba* reached maximum levels at lower salt exposure but stayed unperturbed at higher salt exposures (Figure 3B).

In addition to SOS1-like Na^+^/H^+^ antiporters or other overlapping K^+^/Na^+^ transporters, the HKT category of transporters also take part in Na^+^ exclusion and in maintaining high K^+^/Na^+^ ratio to protect leaves [52]. Our results indicate a weak ability to properly regulate the K^+^/Na^+^ ratio in young as well as old leaves of *E. torquata* (Figure 3C). The K^+^/Na^+^ ratio also decreased significantly in El and Eg, however at a substantially lower magnitude compared to Et, again highlighting a controlled Na^+^ accumulation in Et. Potential deregulation of the SOS regulatory pathway, as appears to be the case, could also add to development of a severe stress phenotype, as SOS signals also regulate cell expansion and cell wall architecture [53] (Figure 3C). Ionic imbalances (K^+^/Na^+^ ratio) influence protein stability and function, pH regulation, membrane integrity, turgor adjustments as well as guard cell function, where K^+^ is required [54,55,56,57]. Experimental evidence in Eucalyptus suggests a positive role of conditional K^+^ fertilization in acclimation to water deprivation [58,59]. Impaired K^+^-Na^+^ homeostasis could affect membrane integrity as was evident for both Eg and Et plants, where electrolyte leakage was substantially higher than El plants (Figure 3E).

Similar findings were reported for the moderately tolerant *E. citriodora* [15], where leaf K^+^ as well as K^+^/Na^+^ ratio dropped on salt exposure [60]. In contrast, salt-tolerant species like *E. spathulate* and *E. sargentii* possess the ability to selectively increase K^+^ amounts over Na^+^ [14], as is the case for *E. loxophleba* in the present study (Figure 3C). The responses of Eg young and old leaves in comparison to El and Et shows a clear example of a moderate response between salt tolerance and sensitivity. Another facet of salt stress is the perturbation of water relations. Ionic imbalance between soil and root affects water uptake, stomatal function and in turn carbon assimilation [27]. Osmotic adjustments in salt-tolerant species could protect against inhibition of normal photosynthetic efficiency. The highlighted moderate response for *E. gomphocephala* was apparent from the increased electrolyte leakage in the sensitive Et (Figure 3D). However, the changes in RWC were similar to the tolerant species El, indicating that RWC is a poorly related parameter (Figure 3D). Further characterization of sub-cellular ion (K^+^, Na^+^ etc.) accumulation and its temporal changes in moderately salinity tolerant/sensitive species like *E. gomphocephala* in contrast to the known tolerant and sensitive species is needed. This could help in finding the accumulation thresholds instrumental in activating the defense signaling pathways under salt stress.

### 3.2. Perturbation in pETC Function

Salinity effects on photosynthesis are manifested by multiple biochemical and physiological parameters; in particular, decreasing stomatal conductance affects gas exchange, K^+^ depletion- and Na-accumulation-induced enzyme inhibition decreases the carboxylation efficiency and oxidative stress affects cell redox homeostasis, metabolism and signaling [4]. The chlorophyll fluorescence-derived data in young leaves of salt-tolerant El indicated marginal stimulation of PSII efficiency in contrast to majorly negative effects in Eg and Et, whereas in old leaves, constitutive values for all calculated parameters were higher in El (than both Eg and Et) with no negative salt effects (Figure 4). This could have contributed to a reduced but sustained growth of these plants without severe stress effects on leaves. Similar findings were also reported in other Eucalyptus studies [61]. In this context, chlorophyll a fluorescence-derived-parameters serve as meaningful indicators for the contrasting stress response in different cultivars/spp of a plant [29,30,62,63]. However, standard measurements are often limited to quantum yield efficiency of PSII only. The recent development of LED array spectrophotometer for in vivo application (DUAL-KLAS-NIR) has improved on this limitation by enabling simultaneous measurements of redox changes in components downstream of PSII in intact leaves [36] and was employed here.

Redox state kinetics of PC, PSI and Fd basically highlight how the state of plant photosynthesis and the rapidity of activation and deactivation of photochemistry differs among leaves along with how abiotic stresses may influence the rates [35,37,38,39]. The observed strong variability among biological replicates for the measurements indicates plasticity of the system to accommodate rapid adjustments that could be needed in response to changing environmental conditions (Figure 5 and Figure 6). However, species-specific differences in the response of PC, PSI and Fd were recorded matching the physiology and biochemical analysis of the stress response (Figure 5 and Figure 6). For example, young leaves of moderately tolerant *E. gomphocephala* plants showed salt-induced changes along with Et especially for PC and PSI (Figure 5). The rate of redox shift and redox state maxima remained relatively constant in El plants. In *E. torquata* plants, the dynamic range of the redox shift was lower, while Eg appeared similar to El at the start of the light phase but ended similarly to Et (Figure 5).

Salt effects were more pronounced in young leaves than old for these measurements. However, more species-dependent differences were visible for old leaves irrespective of strength of salt exposure. *E. loxophleba* plants showed higher flexibility in response to salt in old leaves then *E. torquata,* for example, at 170 mM salt exposure (Figure 6). The higher dynamic range of El plants in old leaves overlapped with constantly higher values for chlorophyll fluorescence-derived PSII efficiency parameters (Figure 4 and Figure 6). It is emphasized that such redox state measurements for PC, PSI and Fd could be useful to detect early signs of salinity tolerance/sensitivity in different plant species. Further, these detailed and deep biochemical features could be applied for molecular breeding in Eucalyptus-like tree species for salinity tolerance traits.

### 3.3. Endomembrane Dynamics in Plant Growth and Stress Acclimation

Subcellular membrane trafficking supports cell wall remodeling, cell proliferation and ion homeostasis among other fundamental processes [64]. Cellular pH homeostasis, ionic fluxes across membranes and hence regulation of H^+^ pumps and cation/H^+^ transporter-like proteins drive membrane trafficking [65]. In this context, salt induced perturbations in K^+^ distribution, selective K^+^/Na^+^-accumulation, nutrient signaling, and pH homeostasis are significant [57,65,66]. Therefore, as has been recently iterated [17], analysis of proteins in vacuoles, which are decisive compartments for Na^+^ accumulation and ion homeostasis, is crucial.

In this study, activities of selected acid hydrolases, functional in acidic compartments of the cell (vacuole, apoplast), generally increased in all Eucalyptus species under salinity (Figure 7). Specifically, activities of phosphatase, phosphodiesterase, and β-galactosidase increased in young and old leaves of *E. loxophleba* (Figure 7). Recorded activity was strongly induced at 80 mM NaCl, but only marginally at 170 mM, indicating activation of protective mechanisms at lower salt concentrations [67] (Figure 7). This pattern was similar for Na^+^ accumulation (Figure 3 and Figure 7). This pattern of increase was generally stronger and unique for El compared to Eg and Et, pointing to potential sturdy endomembrane dynamics and regulation of K^+^-distribution (Figure 3A,C and Figure 7). Such stimulation of membrane trafficking could result in observed changes in vacuolar dimensions for different plant species under abiotic stresses, especially under Cd or salt exposure [68,69,70]. Well-regulated protein activities for selected hydrolases in salt-tolerant species stipulate potential sustenance of such diverse pathways as protein metabolism, membrane functions, transport, stress signaling and cell wall biosynthetic processes for stress acclimation [71,72]. Apparently, these acid hydrolases may serve as sensitive and simple indicators allowing for titrating the salinity response and its saturation, as far as the contribution of the endomembrane dynamics is concerned.

## 4. Materials and Methods

### 4.1. Experimental Setup and Growth under Salinity

Seeds of *E. gomphocephala* A. Cunn. ex DC., *E. loxophleba* Benth., and *E. torquata* Luehm. were planted and grown in plastic pots filled with soil in a green house at 25 ± 2 °C, 150–200 µmol photons m^−2^ s^−1^ light intensity with a long day regime (14/10 h day/night) and 55 ± 2% RH for six months (if not stated otherwise) before exposure to salt stress. They were fertilized using commercial liquid fertilizer WUXAL Super (0.1%) using 80 mL per pot once in two weeks. The composition of 0.1% nutritive solution was N (7.08 mM), P_2_O_5_ (0.35 mM), K_2_O (0.79 mM), B (24 µM), Cu (0.8 µM), Fe (4.5 µM), Mn (2.7 µM), Mo (0.13 µM), and Zn (0.76 µM).

Eucalyptus plants were grown for six months or one year before 30 d of salt exposure (0, 80 and 170 mM NaCl, 30 plants per species, per treatment). Two different growth stages (7 and 13 months) were chosen to analyze the influence of seedling age on salinity-induced growth inhibition. Data presented in figures is labelled suitably when presenting data from both growth stages; otherwise, all other analysis was carried out in 7-month-old plants. For salinity application, the salt amount was progressively raised every second day in two sequential steps i.e., from 80 to 120 to 170 mM. The electrical conductance was measured with the *ECTestr low+* conductivity meter (Oakton, Eutech Instruments Europe B.V., Netherlands) every second day to control the salt concentration in the soil and to adjust its levels by appropriate irrigation or saline solution. After 30 d of salt stress, young and old leaves from different biological replicates were harvested with instant freezing in liquid nitrogen and then stored at 80 °C for further analysis.

### 4.2. Osmotic and Ionic Challenge Posed by Salinity: Measure of Membrane Integrity and Ion Accumulation

Electrolyte leakage, a measure for plasma membrane integrity, was measured for Eucalyptus leaves. Fresh leaves were harvested, washed with deionized water, and made into circular disks of 5 mm diameter. The leaf disks were incubated in glass tubes containing 10 mL of deionized water in a water bath at 32 °C for 2 h and the electrical conductance of the incubation solution was measured (EC 1) with the *ECTestr low+* conductivity meter (Oakton, Eutech Instruments Europe B.V., Netherlands). The tubes were then autoclaved for 20 min to induce maximal loss of ions from cells and electric conductivity measured again (EC 2). Electrolyte leakage (EL) was calculated using Equation (1) [73].

(1) $$\mathrm{EL}\;=\frac{\mathrm{EC}1}{\mathrm{EC}2}*100$$

Relative water content (RWC) was measured with five leaves per sample. After weighing to obtain the actual fresh weight (FM), the leaves were immersed in 100 mL of distilled water for 6 h to obtain the weight of fully turgid leaves (TM). Leaves were dried at 60 °C for 48 h to determine dry weight (DM). The relative water content (RWC) was calculated according to Equation (2) [74]:

(2) $$\mathrm{RWC}\;=\;\frac{\mathrm{FM}-\mathrm{DM}}{\mathrm{TM}-\mathrm{DM}}*100$$

For estimation of ion accumulation, pulverized leaf tissue was extracted in 1 mL of HNO_3_ (1 M) in the Precellys^®^ homogenizer (3 cycles of 6800 rpm, 20 s each with 30 s pause every time). The homogenate was centrifuged at 16,000× *g* for 10 min (4 °C) and supernatant was used for ion contents quantification. Na^+^, K^+^ and Ca^2+^ were determined in leaf extracts using a flame photometer (Model 410; Sherwood Scientific Ltd., Cambridge, UK) [75]. Standard solutions of Na^+^, K^+^ and Ca^2+^ (0–10 ppm) were used to calibrate the flame photometer.

### 4.3. Analysing Salt Stress Effects on Efficiency of pETC

The LED array spectrophotometer (DUAL-KLAS-NIR, Walz, Germany) facilitates measurements of chlorophyll a fluorescence as well as light-driven redox changes in PC, PSI and Fd. The measurement protocol was used as described with slight modifications [38,39]. Young and old leaves of differentially treated plants were detached and incubated in the dark with their petioles placed in water for 90 min before being analysed on the spectrophotometer. For analysis, leaves were fixed between emitter and detector with adaxial leaf surface facing the incident actinic light (photon flux density of 160 µmol m^−2^ s^−1^) or a saturating pulse of light. One standard measurement was 20 s long with an initial 3 s of dark measurement, followed by 17 s of actinic light. Recording was continued for 300 ms after switching off the actinic light.

Recorded chlorophyll a fluorescence data were utilized for calculating the following parameters:

(3) Maximum PSII efficiency in light (FV′/Fm′)=(Fm′−Fo′)Fm′

(4) Operating PSII efficiency in light (Fq′/Fm′)=(Fm′−Ft)Fm′

(5) $$Electron\;transport\;rate\;\left(J\right)=\;(F_{q^{\prime }}/F_{m^{\prime }})\;\times \;\left(160\right)\;\times \;\left(0.5\right)$$

(6) Estimation of open PSII reaction centers (qL)=(Fq′/Fv′)(Fo′/Ft)

where, *F_o’_* = minimum fluorescence in light, *F_m’_* = maximum fluorescence in light, *F_t_* = steady state fluorescence; for *J*, PFD was replaced by incident light assuming similar absorption for all samples, while 0.5 was the factor assumed for equal distribution of light between PSII and PSI. In addition, chlorophyll and carotenoid contents were measured independently in one-year-old plants after a prolonged (2 months) treatment.

For deconvolution of PC, PSI and Fd signals, model spectra generated with the leaves of *E. camaldulensis* were used. It needs emphasis here that *E. camaldulensis* is a well-recognized salt-tolerant Eucalyptus species and has been used for deconvolution here in all the salt treated plants for uniformity of background correction. Further, along with chlorophyll a fluorescence and light-driven redox changes for PC, PSI and Fd, another automated script, i.e., NIR_max_ allowed measurement of maximum redox change for PC, PSI and Fd and was used to scale the redox shift to obtain absolute values as well as pool size ratios for PC and Fd relative to PSI. NIR_max_ measurements required dark adaptation. The kinetics of light-driven redox change here was divided into five phases as described in the figure legend.

### 4.4. Enzyme in Antioxidant Defense and Endomembrane Metabolism: Testing Salt Stress Effects

Salt stress due to ionic accumulation may lead to generation of ROS or inhibit enzyme activities/protein function in the target organelle. In the present setup, activities of the H_2_O_2_ scavenging enzyme catalase and the endomembrane system-associated vacuolar hydrolases phosphatase, phosphodiesterase and β-galactosidase were measured in all Eucalyptus spp. For protein extraction, fresh leaves were pulverized in liquid N_2_ and homogenized (±100 mg) in 500 µL of K-Pi buffer (50 mM; pH 6.8) containing 1 mM phenylmethylsulfonylfluoride (PMSF) as protease inhibitor, using Precellys^®^ homogenizer (3 cycles of 6800 rpm, 20 s each with 30 s pause every time). The homogenate was centrifuged at ~16,000× *g* for 15 min (4 °C) and the cleared extract was used for the assay. Protein content was determined spectrophotometrically at 595 nm using the Bradford assay [76].

Catalase activity was determined polarographically with a Clark-type O_2_ electrode [77]. The assay mixture contained K–Pi buffer (50 mM; pH 6.8) and 10 µL extract, where after obtaining the baseline, the assay was started by adding H_2_O_2_ (40 mM). Catalase-specific activity was presented as nmol min^−1^ µg^−1^ protein. Activity of hydrolytic enzymes phosphatase, phosphodiesterase and β-galactosidase was measured by determining the liberation of p-nitrophenol (PNP) from PNP-phosphate, bis-PNP-phosphate and PNP-β-galactopyranoside, respectively [78]. A fixed volume of protein extract was added to the reaction mixture (400 µL) of citric acid-KOH (100 mM; pH 4.6) and 2 mg of respective PNP substrate. After incubation for 60 min at 37 °C, the assay was stopped by adding 140 µL Na_2_CO_3_ (1 M), and the absorption was measured at 405 nm. Turnover rate was calculated using the molar extinction coefficient of p-nitrophenol (ε = 18.5 mM^−1^ cm^−1^). 

### 4.5. Statistical Analysis

Values presented are means ± SE, if not mentioned otherwise. The data were analyzed for significance of difference using IBM SPSS Statistics 21 software. Data were subjected to analysis of variance (ANOVA) followed by post-hoc analysis (Tukey’s test; *p* < 0.05 level). Means not labelled with letters show no statistically significant differences. Plant height and leaf elongation were measured every 5th day. The values for leaf size and plant height were normalized in a way to highlight changes during the 30 d stress. For this, the values collected before treatment were subtracted from all values recorded during treatment duration and data presented in Figure 1 and Figure 2. To emphasize the observed differences in growth, trend-lines are shown for normalized data in both figures.

## 5. Conclusions

The efficient ionic and osmotic balance maintenance in El plants highlights the salinity tolerance feature of this Eucalyptus species, in contrast to moderate or low salt tolerance as observed for Eg and Et species, respectively. The early response to salt exposure shown by hydrolytic enzymes, especially in the tolerant species El, further highlights the importance and need for detailed investigation into the dynamic nature of the vacuole and apoplast function under salinity. The results here show two crucial aspects of pETC functioning: (a) the variability of the constitutive response for these Eucalyptus species to dark-light transitions, and (b) the impact of the salt treatment. Since the first response was as equally prominent as the salinity effects, it was tedious to distinguish the two in certain cases. This further highlights that the observed differences have implications for not only salinity stress, but water deprivation in general, due to their direct influence on photosynthesis.

## Data Availability

Data are contained within the article and supplementary materials.

## Supplementary Materials

The following are available online at https://www.mdpi.com/article/10.3390/plants10071401/s1, Figure S1: Comparison of accumulated biomass (fresh and dry weight of leaves, stem, and roots) for optimally grown *E. gomphocephala*, *E. loxophleba* and *E. torquata*, Figure S2: Leaf pigment contents of *E. gomphocephala*, *E. loxophleba* and *E. torquata* as affected by salt treatment, Figure S3: Ca^2+^-contents of leaf tissue from salt exposed *E. gomphocephala*, *E. loxophleba* and *E. torquata*, Figure S4: Catalase activity of *E. gomphocephala*, *E. loxophleba* and *E. torquata* leaves under salinity stress, Table S1: Germination of multiple Eucalyptus spp from Tunisia exposed to increasing concentrations of salt (0–210 mM NaCl), Table S2: Root and shoot growth data from multiple Eucalyptus spp exposed to increasing concentrations of salt (0–210 mM NaCl), Table S3: Maximum redox change and ratio of pool sizes for PC, P700 and Fd in salt-treated Eucalyptus spp (*E. gomphocephala*, *E. loxophleba* and *E. torquata*) estimated after dark adaptation using DUAL-KLAS-NIR spectrophotometer.
