# Supplementary material for: Differences in Ionic, Enzymatic, and Photosynthetic Features Characterize Distinct Salt Tolerance in Eucalyptus Species
Source: Plants (Basel). 2021 Jul 9;10(7):1401. doi: 10.3390/plants10071401 (PMC8309277; doi:10.3390/plants10071401)
Supplement: Supplementary file 1 [file plants-10-01401-s001.zip › Figures S1-S4.pdf]

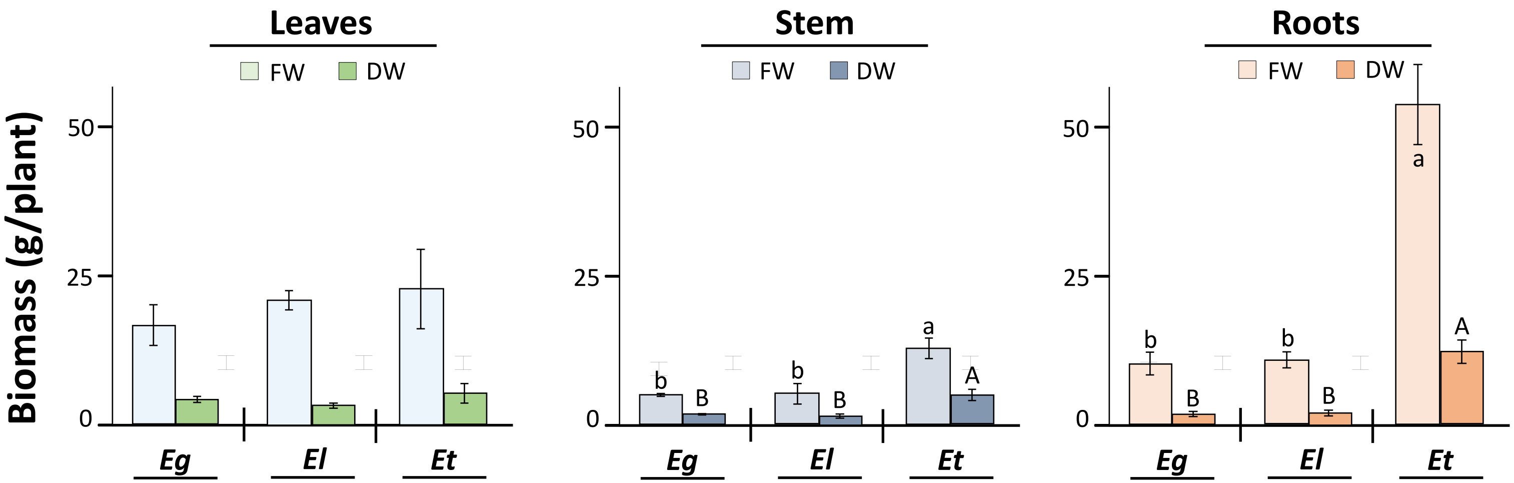

Figure S1: Comparison of accumulated fresh and dry weight (leaves, stem, and roots) for optimally grown *E. gomphocephala*, *E. loxophleba* and *E. torquata*. Selected *Eucalyptus* spp. (*E. gomphocephala*, *E. loxophleba* and *E. torquata*) were compared for there biomass accumulation (fresh weight; FW and dry weight; DW) under optimal growth conditions after one year of growth. Data are means ± SE, n=3 ($p<0.05$; ANOVA and Tukey's post hoc test; a, b, c…for FW and A, B, C…for DW).

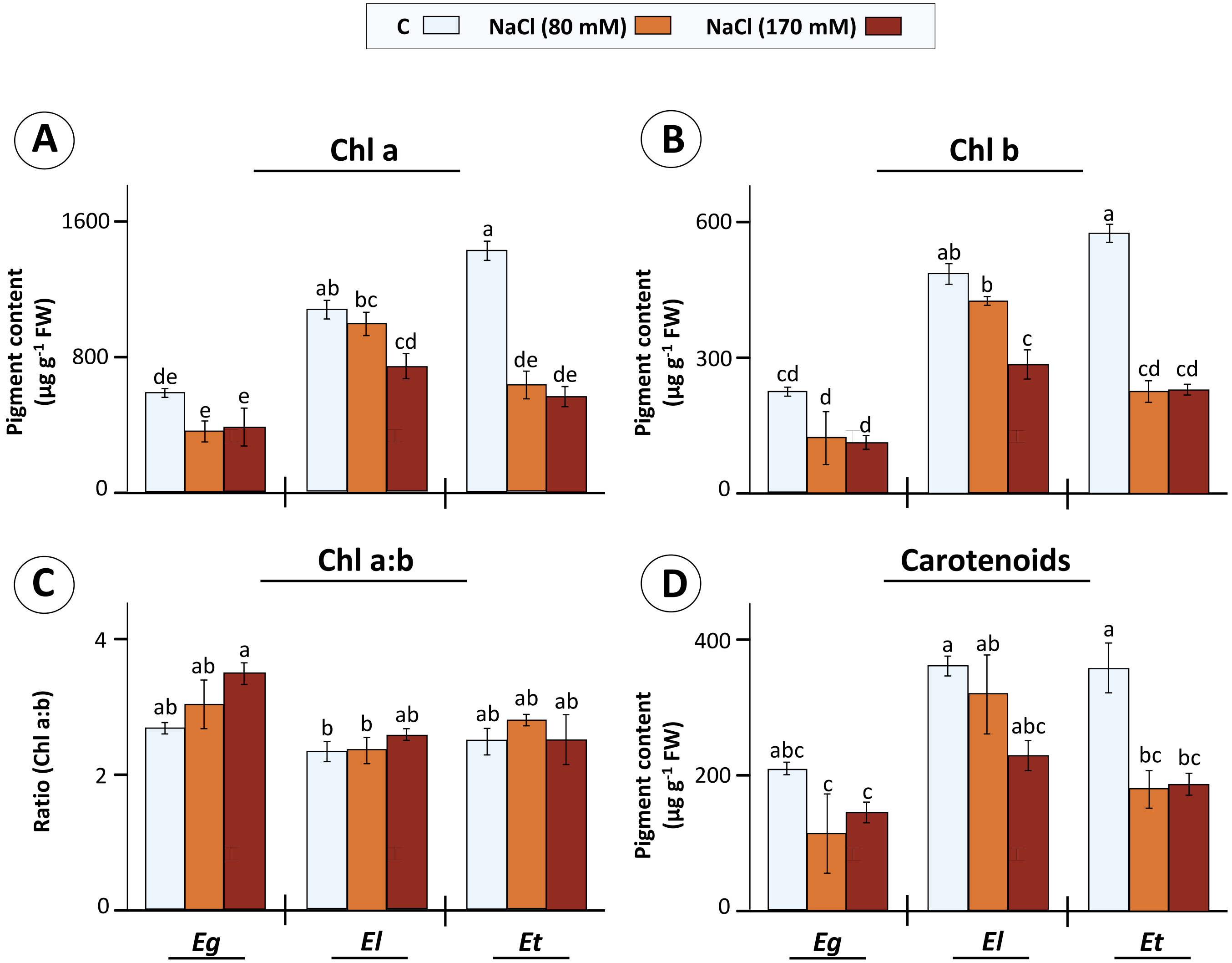

Figure S2: Pigment contents measured in *E. gomphocephala*, *E. loxophleba* and *E. torquata* as affected by salt treatment. Chlorophyll (A, B, and C) and carotenoid (C) contents were measured in leaves of salt treated Eucalyptus plants after one year growth under optimal conditions. Data are means ± SE, n=3 ($p<0.05$; ANOVA and Tukey's post hoc test).

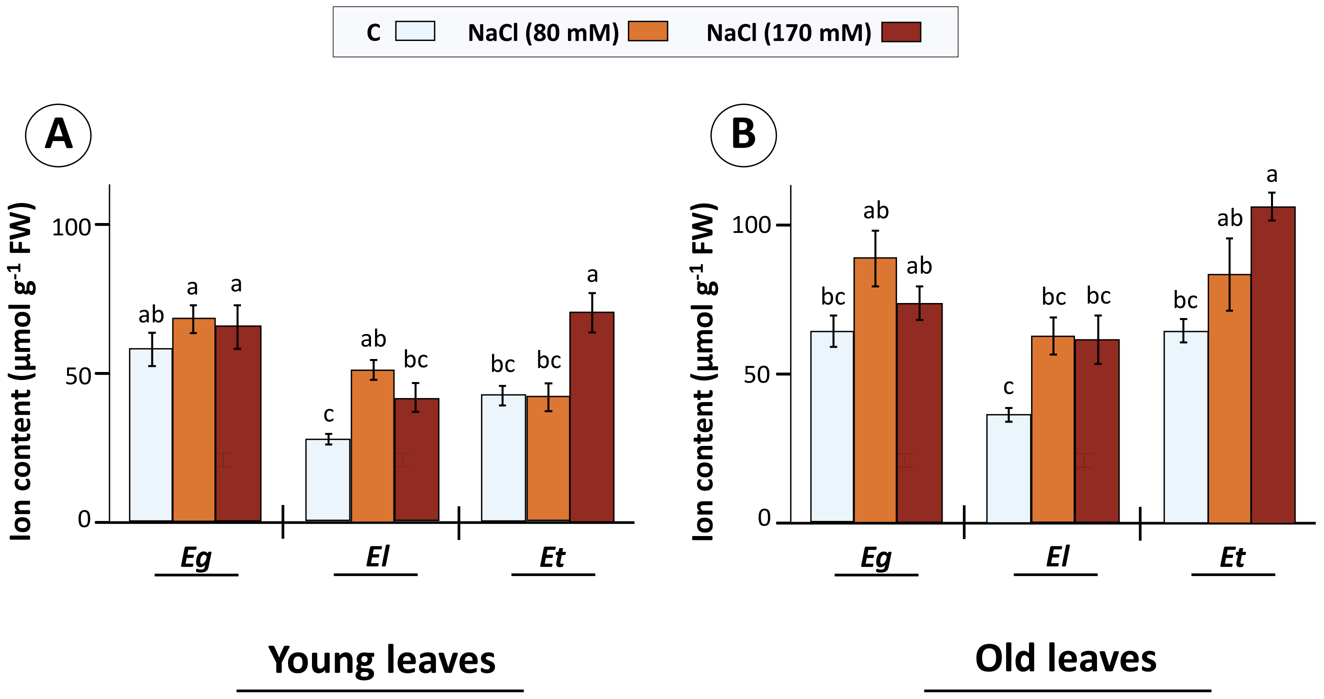

Figure S3: Ca^{2+}-contents measured in leaf tissue of salt exposed *E. gomphocephala*, *E. loxophleba* and *E. torquata*. Selected Eucalyptus plants were grown for 6 months before exposure to NaCl (80 and 170 mM) for 30 d. Ca^{2+}contents were measured after the treatment period in the young (A) and old (B) leaf tissues. Data are means ± SE, n=5 ($p<0.05$; ANOVA and Tukey's post hoc test).

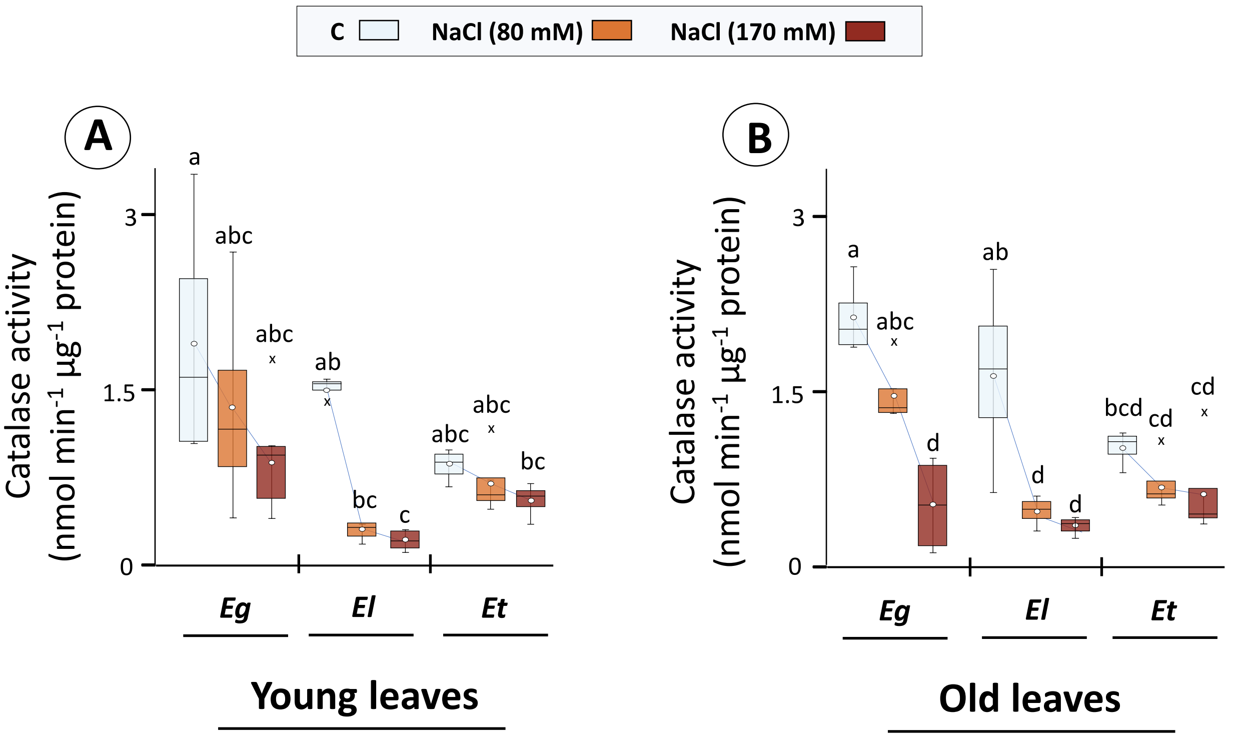

Figure S4: Catalase activity measured in *E. gomphocephala*, *E. loxophleba* and *E. torquata* leaves under salinity stress. Activity of the H_2O_2 scavenging enzyme catalase was measured in young (A) and old leaves (B) of 30 d salt (80 or 170 mM NaCl)-treated Eucalyptus spp after 6 months of optimal growth. Data are presented in box plots where the hollow circle shows the position of mean, the horizontal line in the box shows median, box limits indicate the 25th and 75th percentiles, whiskers extend 1.5 times the interquartile range from the 25th and 75th percentiles, while not connected data points (outliers) are presented by cross. Statistics results are presented to compare means (n=4, p<0.05; ANOVA and Tukey's post-hoc test).
